# Alzheimer-Erkrankung – welche Substanzen befinden sich derzeit in Phase-III-Studien?

**DOI:** 10.1007/s00115-022-01275-5

**Published:** 2022-03-14

**Authors:** Klaus Hager

**Affiliations:** Institut für Allgemeinmedizin und Palliativmedizin, Medizinische Hochschule Hannover (MHH), Carl-Neuberg-Str. 1, 30625 Hannover, Deutschland

**Keywords:** Demenz, Zukünftige Therapie, Kognition, Kranheitsverändernd, Beta-Amyloid, Dementia, Future therapy, Cognition, Disease modifying, Amyloid-beta

## Abstract

**Hintergrund:**

Die kürzlich von der US Food and Drug Administration (FDA) zugelassene Substanz Aduhelm/Aducanumab schürt die Hoffnung, dass weitere Substanzen gegen die Alzheimer-Erkrankung vor der Zulassung stehen könnten.

**Ziel der Arbeit:**

Ziel war es, den Stand der derzeitigen Phase-III-Studien zur medikamentösen, auf die Verbesserung der Kognition gerichteten Therapie bei Alzheimer-Erkrankung aufzuzeigen.

**Material und Methoden:**

Die US-amerikanische Datenbank, www.clinicaltrials.gov, wurde 07.08.2021 nach aktuellen Studien zur Alzheimer-Erkrankung durchsucht und diejenigen Phase-III-Studien weiter ausgewertet, deren primärer Endpunkt eine Verbesserung der Kognition war. Das Wirkprinzip der Substanzen wurde nach der Common Alzheimerʼs Disease Research Ontology (CADRO) klassifiziert.

**Ergebnisse:**

Von den gefundenen 53 Studien bezogen sich 32 Studien mit 25 Substanzen auf die Beeinflussung der Kognition, kenntlich an einem entsprechenden Test als einem der primären Endpunkte. Zwanzig der Studien sind derzeit aktiv und rekrutieren, 4 weitere rekrutieren noch nicht und 8 nehmen derzeit keine Patienten auf. Sieben Studien zielen auf Beta-Amyloid sowie Tau. Bei 20 Substanzen wird eine die Krankheit verändernde Wirkung postuliert. Jeweils 8 der Studien werden voraussichtlich 2022 und 2023 beendet sein.

**Diskussion:**

Das Spektrum der Wirkmechanismen der Substanzen, die sich derzeit in Studien befinden, erscheint breit und hat überwiegend eine Veränderung des Krankheitsverlaufs zum Ziel. In den kommenden 2 Jahren besteht daher die Möglichkeit, dass weitere Medikamente gegen die Alzheimer-Erkrankung zugelassen werden könnten.

## Abstract (en)

**Background:**

The recent approval of aduhelm/aducanumab by the US Food and Drug Administration (FDA) in June 2021 raised hopes that further substances against Alzheimer’s disease might be approved in the near future.

**Objective:**

The current status of phase III studies for Alzheimerʼs disease was evaluated.

**Material and methods:**

The American database www.clinicaltrials.gov was searched on 7 August 2021 for phase III studies which target the cognitive functions affected by Alzheimerʼs disease. The mode of action of the different substances was classified according to the Common Alzheimerʼs Disease Research Ontology (CADRO).

**Results:**

With the applied search criteria 53 studies were found, 32 studies with 25 substances dealt with cognition, which was demonstrated by a cognition test as one of the primary outcome measures, 20 of the studies are recruiting, 4 are not yet recruiting and 8 studies are active but not recruiting. Of the studies seven target amyloid beta-peptide as well as tau protein. For 20 substances a disease modifying action is assumed. In 2022 and 2023 a total of 8 studies each are planned to be terminated.

**Conclusion:**

The mode of action of the substances, which are currently in studies, seems broad and for their action a modification of the disease is mostly assumed. Therefore, there is hope that in the next 2 years further drugs against Alzheimerʼs disease might be approved.

## Hintergrund

Die Alzheimer-Erkrankung ist eine im Verlauf mit einer schweren Funktionsbeeinträchtigung und früherem Tod einhergehende neurodegenerative Erkrankung, die sich anfangs unbemerkt über eine präklinische Phase von 10 bis 20 Jahren erstreckt, dann in eine mehrjährige prodromale Phase mit geringen Zeichen mündet, bis sie schließlich in die klinische Phase der Alzheimer-Demenz (AD) übergeht. Eine medikamentöse Therapie der Alzheimer-Erkrankung, die diesen Verlauf aufhält oder gar umkehrt, gab es trotz großer Bemühungen bis vor Kurzem nicht. Überhaupt hat die medikamentöse Therapie der Erkrankung in den vergangenen 20 Jahre wenig Fortschritt erfahren. Durch die Zulassung der US Food and Drug Administration (FDA) von Aduhelm/Aducanumab gibt es seit Juni 2021 allerdings einen Hoffnungsschimmer.

## Ziel

In diesem Zusammenhang stellt sich die Frage, welche weiteren Substanzen gegen die Alzheimer-Erkrankung und insbesondere zur Verbesserung der Kognition sich noch in der Prüfung befinden und ob in der nächsten Zeit vielleicht mit weiteren Zulassungen gerechnet werden könnte.

## Methode

Die Datenbank www.ClinicalTrials.gov der National Library of Medicine und des National Institute of Health (NIH) der USA wurde am 07.08.2021 durchsucht. Die Suchkriterien waren: „Phase III“, „recruiting“, „not yet recruiting“, „active, not recruiting“ und „Alzheimer“. Es wurde dagegen nicht nach „suspended“, „terminated“ oder „completed“ gesucht.

Die angenommene Wirkung der Substanzen wird nach der Klassifikation der Common Alzheimerʼs and Related Dementias Research Ontology (CADRO) geordnet [1]. Weiterhin werden die Substanzen entweder einer mutmaßlich nur symptomatischen oder einer krankheitsverändernden Wirkung zugeordnet.

## Resultate

Mit den geschilderten Suchkriterien wurden 53 Studien gefunden, 32 davon bezogen sich auf die Kognition (Tab. 1). Dies war dadurch definiert, dass der primäre Erfolgsparameter einen Test der Kognition beinhalten sollte. Studien, die sich mit psychiatrischen Fragestellungen (Agitation) oder beispielsweise mit der Lebenssituation, dem Einfluss von Diät oder Licht befassten, wurden nicht in die Auswertung einbezogen (Tab. 1). Die 32 Studien mit einem primären Erfolgsparameter im Bereich der Kognition wurden weiter analysiert. Sie umfassten 25 unterschiedliche Substanzen (Tab. 2). Ein Teil der Phase-III-Studien rekrutiert noch nicht, ein weiterer Teil der Studien ist zwar noch aktiv, rekrutiert aber nicht mehr. Zwanzig Studien befinden sich in der Rekrutierungsphase.

**Table 1:** 

Phase	Anzahl der Treffer
*Phase III (insgesamt)*	53
Kognition	32
Rekrutiert noch nicht	4
Aktiv, rekrutiert	20
Aktiv, rekrutiert nicht	8
Agitation	11
Lebenssituation, Diät, Licht und andere	10
*Phase II*	158
*Phase I*	104

**Table 2:** 

	Substanz	Studienakronym	Wirkungsprinzip	Probanden, Kognition	Primärer Erfolgsparameter	Studienstatus	Beginn/Ende^b^	Firma/Sponsor
1	AGB101	HOPE4MCI	Levetiracetam, Antiepileptikum, SV2A-Modulator	MCI	CDR-SB	Aktiv, rekrutiert nicht	2019/2022	AgeneBio, Inc
2	ALZ-801	APOLLOE4	Soll Aggregation von Beta-Amyloid zu Oligomeren hemmen	Apo E4 Homozygote mit milder AD	ADAS-COG	Rekrutiert	2021/2024	Alzheon Inc
3	ANAVEX2-73-AD-004	–	Blarcamesine, selektiver SIGMA-1-Rezeptor-Agonist	MCI, initiale AD	ADAS-Cog and ADCS-ADL	Rekrutiert	2018/2022	Anavex Life Science Corp
4	ANAVEX2-73	ATTENTION-AD	Offene Phase von ANAVEX2-73-AD-004	MCI, initiale AD	Sicherheit und Verträglichkeit	Rekrutiert	2019/2024	Anavex Life Science Corp
5	BDPP	BDPP	Resveratrol, Nahrungsergänzungsmittel	MCI und Prädiabetes bzw. Typ-II-Diabetes	Mehrere	Rekrutiert	2015/2022	Johns Hopkins University
6	BHV-4157	T2 Protect AD	Troriluzole, Glutamatantagonist, Prodrug von Riluzole	Mild bis moderat	ADAS-Cog, CDR-SB	Aktiv, rekrutiert nicht	2018/2020	Biohaven Pharmaceuticals
7	BPDO-1603	BPS 034	Unklar	Moderate bis schwere AD	SIB, CIBIC	Rekrutiert	2020/2023	Hyundai Pharmaceutical
8	Coffein	CAFCA	Stimulierende Eigenschaften	Milde bis moderate AD	NTB	Rekrutiert noch nicht	2021/2024	University Hospital, Lille
9	COR388	GAIN	Atuzaginstat, Inhibitor der Proteasen von *Porphyromonas gingivalis* (Parodontitiserreger)	Milde bis moderate AD	ADAS-Cog1, ADCS-ADL	Aktiv, rekrutiert nicht	2019/2022	Cortexyme, Inc
10	Donanemab	TRAILBLAZER-ALZ 2	Monoklonaler Antikörper gegen Beta-Amyloid	Beginnende symptomatische AD	iADRS	Rekrutiert	2020/2023	Eli Lilly
11	Donepezil	CHOLINE‑2	Acetylcholinesterasehemmer (AChI)	Neu diagnostizierte AD	MMSE	Rekrutiert noch nicht	2021/2024	Hôpitaux de Paris
12	Gantenerumab	GRADUATE 1	Monoklonaler Antikörper gegen β‑Amyloid, subkutane Gabe	Prodromale bis milde AD	CDR-SOB	Rekrutiert	2018/2023	Hoffmann-La Roche
13	Gantenerumab	GRADUATE 2	Monoklonaler Antikörper gegen Beta-Amyloid, subkutane Gabe	Prodromale bis milde AD	CDR-SOB	Aktiv, rekrutiert nicht	2018/2023	Hoffmann-La Roche
14	Gantenerumab	ClinicalTrials.gov ID: NCT04374253	Open-label, Multicenter-Rollover-Studie	Prodromale bis milde AD	CDR-SOB	Rekrutiert	2018/2023	Hoffmann-La Roche
15	Gantenerumab	ClinicalTrials.gov ID: NCT04339413	Open-label, Multicenter-Rollover-Studie	Prodromale bis milde AD	CDR-SOB	Aktiv, rekrutiert nicht	2018/2023	Hoffmann-La Roche
16	Guanfacine	NorAD	Alpha-2-Rezeptor Agonist	AD	ADAS-Cog	Rekrutiert	2019/2022	Imperial College London
17	GV-971^a^	GREEN MEMORY	Wirkung unklar, Oligosaccharide aus Algen	Mild bis moderat	ADAS-Cog, ADCS-CGIC	Rekrutiert	2020/2025	Shanghai Green Valley Pharm
18	Hydralazin	EHSAN	Vermuteter antineurodegenerativer Effekt	Milde AD	ADAS-Cog	Rekrutiert noch nicht	2021/2023	Shahid Sadoughi University
19	Lecanemab	Clarity AD	Monoklonaler Antikörper gegen Beta-Amyloid	Frühe Alzheimer-Erkrankung (EAD)	CDR-SB	Rekrutiert	2019/2024	Biogen, Eisai
20	Lecanemab	AHEAD 3‑45	Monoklonaler Antikörper gegen Beta-Amyloid	Präklinische AD	CDR-SB	Rekrutiert	2020/2027	Biogen, Eisai
21	Losartan + Amlodipin + Training	rrAD	Aerobes Training und intensive vaskuläre Risikoreduktion	Alte Menschen mit Risiko für AD	ADCS-PACC, NIHTB-CB	Aktiv, rekrutiert nicht	2017/2022	University of Texas
22	Metformin	MAP	Antidiabetikum	Amnestisches MCI	FCSRT	Rekrutiert	2021/2025	Columbia University
23	NE3107	–	Antidiabetikum, Entzündungshemmer	Mild bis moderat	ADAS Cog12, ADCS CGIC	Rekrutiert	2021/2023	BioVie
24	Octohydroaminoacridine	–	Acetylcholinesterasehemmer	Mild bis moderat	ADAS-Cog	Rekrutiert	2017/2021	Shanghai Mental Health Center
25	Omega-3-Fettsäure	LO-MAPT	Nahrungsergänzung, mehrfach ungesättigte Fettsäuren	Gedächtnisstörungen od. fam. Belastung	Score: FCSRT, MMSE, CNT	Rekrutiert	2018/2023	Univ. Hospital, Toulouse
26	Semaglutide	EVOKE	Ozempic, Antidiabetikum, GLP-1-Rezeptor-Agonist	MCI, initiale AD	CDR-SB	Rekrutiert	2021/2025	Novo Nordisk A/S
27	Semaglutide	EVOKE Plus	Ozempic, Antidiabetikukm, GLP-1-Rezeptor-Agonist	Frühe AD	CDR-SB	Rekrutiert	2021/2025	Novo Nordisk A/S
28	Simufilam	RETHINK-ALZ	Bindet an Filamin A, hemmt Tau-Hyperphosphorylierung	Mild bis moderat	ADAS-Cog12, ADCS-ADL	Rekrutiert noch nicht	2021/2023	Cassava Sciences
29	Solanezumab, Gantenerumab	DIAN-TU	Monoklonaler Antikörper gegen Beta-Amyloid	Dominant vererbte Alzheimer-Erkrankung	DIAN-MCE	Rekrutiert	2012/2022	Washington Univ. School of Med
30	Solanezumab	A4	Monoklonaler Antikörper gegen Beta-Amyloid	Ältere Probanden mit Risiko für eine AD	PACC	Aktive, rekrutiert nicht	2014/2022	Eli Lilly and Company
31	Tricaprilin	MRCT	Nahrungsergänzungsmittel	Mild bis moderat bis schwer	ADAS-Cog	Rekrutiert noch nicht	2022/2024	Cerecin Pte. Ltd
32	TRx0237	–	Tau-Protein-Aggregationshemmer	Prodromale bis milde AD	ADAS-cog, ADCS-ADL	Aktiv, rekrutiert nicht	2018/2023	TauRx Therapeutics Ltd

*AD* Alzheimer Demenz, *ADAS-COG* Alzheimer’s Disease Assessment Scale – Cognitive Subscale, *ADCS-ADL* Alzheimer’s Disease Cooperative Study – Activities of Daily Living, *ADCS-CGIC* Alzheimer’s Disease Cooperative Study-Clinical Global Impression of Change, *ADCS-PACC* Alzheimer’s Disease Cooperative Study-Preclinical Alzheimer Cognitive Composite, *CDR-SB* Clinical Dementia Rating Scale Sum of Boxes, *CIBIC* Clinician Interview-Based Impression of Change, *CNT* Category Naming Test, *DIAN-MCE* Dominantly Inherited Alzheimer Network-Multivariate Cognitive Endpoint, *PACC* Preclinical Alzheimer Cognitive Composite; *FCSRT* Free and Cued Selective Reminding Test, *iADRS* integrated Alzheimer’s Disease Rating Scale (ADAS-Cog13 und ADCS-iADL), *MCI* mild cognitive impairment, *MMSE* Mini Mental Status Examination, *NIHTB-CB* NIH Toolbox Cognition Battery, *NTB* Neuropsychological Test Battery, *SIB* Severe Impairment Battery

^a^In China seit 2019 für leichte bis moderate Alzheimer-Erkrankung zugelassen [12]

^b^„Actual Study Start Date/Estimated Study Completion Date“

Die Zahl der Studien in Phase I und II zeigt, dass „Nachschub“ an Phase-III-Studien vorhanden ist.

Die Studien werden anhand der Angaben aus der Datenbank in einer Tabelle zusammengefasst (Tab. 2). Die Vielfalt der geprüften Substanzen ist erst einmal verwirrend. Daher wurden sie nach dem mutmaßlichen Wirkprinzip geordnet (Tab. 3). Neben den schon geläufigen Wirkungen hinsichtlich Neurotransmitter (z. B. Hemmung der Acetylcholinesterase), der Wirkung auf Beta-Amyloid (z. B. monoklonale Antikörper) und der Wirkung auf die Tau-Pathologie finden sich auch Entzündungshemmer, Antioxidanzien, Antidiabetika oder Nahrungsergänzungsmittel unter den getesteten Substanzen. Eine etwas ungewöhnliche Substanz ist das Atuzaginstat (COR388, GAIN-Studie). Dabei handelt es sich um einen Inhibitor der Proteasen Lysin- und Arginin-Gingipain des Bakteriums *Porphyromonas gingivalis*, einem Parodontitiserreger. *Porphyromonas gingivalis* und Gingipaine wurden auch im Gehirn von Alzheimer-Patienten gefunden.

**Table 3:** 

Ziel	Substanzen
Beta-Amyloid	Solanezumab (KV), Gantenerumab (KV), Lecanemab (KV), Donanemab (KV), ALZ-801 (KV)
Tau	TRx0237 (KV), Simufilam (KV)
Neurotransmitterrezeptoren	Donepezil (S), Guanfacine (S), Octohydroaminoacridine (S)
Entzündung/Infektion	COR388 (KV), NE3107 (KV)
Oxidativer Stress	BDPP (KV), Omega-3-Fettsäure (KV)
Metabolismus und Bioenergetik	Tricaprilin (KV), Metformin (KV), Semaglutide (KV), Coffein (S)
Gefäße	Losartan + Amlodipin + Training (KV), Hydralazin (KV)
Synaptische Plastizität/Neuroprotektion	ANAVEX2-73 (KV), AGB101 (KV), BHV-4157 (KV)
Darm-Gehirn-Achse	GV-971 (KV)
Unbekannt	BPDO-1603 (unbekannt)

*KV* krankheitsverändernd; *S* symptomatisch

Unter der Überlegung, dass Behandlungsmöglichkeiten am ehesten bei der initialen Alzheimer-Erkrankung wirksam sein werden, schließen die Studien ganz überwiegend Patienten mit prodromaler bis milder bzw. initialer Alzheimer-Demenz ein. Als primärer Erfolgsparameter wurde in vielen Studien die Clinical Dementia Rating Scale Sum of Boxes (CDR-SOB) gewählt, welche die vor einigen Jahren noch häufiger verwendete Alzheimer’s Disease Assessment Scale-Cognitive Subscale (ADAScog) als Erfolgsparameter zu verdrängen scheint. Bedingt durch die hohen Kosten einer Phase-III-Studien sind die Studieninitiatoren vor allem Pharmafirmen, selten beispielsweise Universitäten (Tab. 2). Der überwiegende Teil der Substanzen zielt auf einen krankheitsverändernden Verlauf ab (Tab. 3).

## Diskussion

Es gibt eine große Zahl von Studienregistern, z. B. das Clinical Trial Registry der Europäischen Union [10]. Sie sind über die International Clinical Trials Registry Platform (ICTRP) der Weltgesundheitsorganisation (WHO; [2]) auffindbar. Da der größere Teil der Studien zur Demenz in den USA initiiert wird, wurde das amerikanische Register www.ClinicalTrials.gov für die Auswertung herangezogen.

In Anbetracht der Zeit, die zwischen einer Phase-I- oder -II-Studie und der eventuellen Zulassung vergeht, wurden nur die Phase-III-Studien ausgewertet. Auflistungen über alle Phasen finden sich jedoch in der Literatur (z. B. [5, 7]). Hierzu wurden 53 Studien vorgefunden. Davon wurden nur diejenigen weiter betrachtet, bei denen eine Verbesserung der geistigen Leistung ein primäres Studienziel war. Studien zu Verwirrtheit, Biomarkern oder anderen Ergebnissen wurden hier nicht betrachtet. Nach dieser Einschränkung ergaben sich 25 Substanzen in 32 Studien. Etwas über 250 Studien befinden sich derzeit in der Phase I und II. Aus den Angaben in der Datenbank wurden die Studien in einer Tabelle angeordnet, aus der unter anderem die Substanz, der Status der Studie und das voraussichtliche Enddatum hervorgehen (Tab. 2). Eine jährlich durchgeführte Analyse dieser Art ließe einen Trend zum Schwerpunkt der Studientätigkeit erkennen [7].

Die einzuschließenden Probanden sollten überwiegend eine prodromale bis milde Alzheimer-Demenz aufweisen. Nur eine der Studien lässt auch eine schwerere Form der AD zu (MRCT mit Tricaprilin), was die aktuelle Auffassung widerspiegelt, dass initiale Formen einer Therapie besser zugänglich sind.

Für die Diagnose einer Alzheimer-Erkrankung werden zumeist positive Biomarker (z. B. Liquorpunktion, Amyloid-PET) gefordert. Eine Veränderung der Biomarker unter der Therapie wäre ein gutes Argument für eine den Krankheitsverlauf verändernde Wirkung und ein Vorteil bei der Zulassung.

Als Status der Studien wird in der Datenbank „not yet recruiting“, „recruiting“ sowie „active, not recruiting“ angegeben. „Recruiting“ zeigt an, dass die Studie läuft und Probanden eingeschlossen werden. Der Grund, warum eine Studie aktiv ist, jedoch nicht mehr rekrutiert, kann variieren. So hat die GAIN-Studie (Tab. 2) die vorgesehene Zahl von 643 Probanden rekrutiert und nimmt daher keine weiteren Probanden auf. Zusätzlich wurde von der FDA die unverblindete Fortführung der Studie in einer „open label extension“ (OLE) abgebrochen, da hepatische Nebenwirkungen beobachtet wurden. Der Status „not yet recruiting“ wird in erster Linie deshalb bestehen, weil die betreffende Studie noch nicht bei der vorgesehenen Probandenrekrutierung angelangt ist.

Die postulierten Wirkungsweisen der gefundenen Substanzen werden nach der Klassifikation CADRO geordnet [1]. Die Klassifikation bietet 18 Wirkweisen an, in die die Medikamente eingeordnet werden können. In Tab. 3 sind hiervon nur diejenigen aufgeführt, denen tatsächlich Medikamente zugeordnet werden konnten. Die Einstufung ist nicht immer eindeutig und erfolgte daher überwiegend nach der Literatur [5]. In manchen Teilen bleibt die Eingruppierung spekulativ. So wurden die Studien mit Omega-3-Fettsäuren dem Bereich „oxidativer Stress“ zugeordnet [5]. Andere Kategorien wären ebenfalls denkbar gewesen. Die Unsicherheit wird auch dadurch deutlich, dass die Autoren das Wirkprinzip 2020 noch bei „synaptischer Plastizität/Neuroprotektion“ sahen [4], 2021 aber bei „oxidativem Stress“ [5].

Am häufigsten sind die Amyloidablagerungen Ziel der Studiensubstanzen und dabei vor allem monoklonale Antikörper wie Solanezumab, Donanemab, Gantenerumab oder Lecanemab (Tab. 2). Im Verlauf der letzten Jahre ist allerdings der Anteil der Anti-Amyloid-Studien etwas rückläufig [7].

Im Zusammenhang mit der Wirkungsweise ist die Frage interessant, welche der Substanzen den Verlauf der Alzheimer-Erkrankung verändern könnten und welche nur symptomatisch wirken (Tab. 3). Die hier getroffene Zuordnung zu „symptomatisch“ oder „krankheitsverändernd“ folgt erneut überwiegend nach Angaben aus der Literatur [5]. Eine krankheitsverändernde Therapie wird am ehesten von den monoklonalen Antikörpern gegen Beta-Amyloid zu erwarten sein, die bereits im Tierexperiment sowie in Studien am Menschen nachweisen konnten, dass sie die Amyloidmenge im Gehirn reduzieren können (z. B. [11]).

Die Datenbank gibt auch ein geschätztes Studienende an (Tab. 4), wobei es natürlich zu Verschiebungen kommen kann, z. B. da die Rekrutierung der Probanden länger als geplant dauert.

**Table 4:** 

Zeitpunkt	Substanz
2021	Octohydroaminoacridine
2022	AGB101, ANAVEX2-73-AD-004, BDPP, COR388, Guanfacine, Losartan + Amlodipin + Training, Solanezumab + Gantenerumab, Solanezumab
2023	BPDO-1603, Donanemab, Gantenerumab, Hydralazin, NE3107, Omega-3-Fettsäure, Simufilam, TRx0237
2024	ALZ-801, ANAVEX2-73, Coffein, Donepezil, Lecanemab, Tricaprilin
2025	GV-971, Metformin, Semaglutide
2027	Lecanemab

Die Abkürzungen ergeben sich aus Tab. 2

*2020, 2021:* Die T2-Protect-AD-Studie mit BHV-4157 (Troriluzole) sollte plangemäß 2020 abgeschlossen werden. In einer Pressemitteilung der Firma Biohaven wurde im Januar 2021 gemeldet, dass die beiden primären Endpunkte keinen Unterschied zur Placebogabe aufwiesen, die Studie also nicht erfolgreich verlaufen war [3].

In Februar 2021 sollte die Studie mit Octohydroaminoacridine beendet sein, ein Medikament, das in China bereits seit 2019 für leichte bis moderate Alzheimer-Erkrankung zugelassen ist [12]. Die Studie ist aber offensichtlich noch nicht abgeschlossen.

Bei der Studie mit COR388 (GAIN) wird für das Studienende das Jahr 2022 angegeben, Resultate wurden im Internet aber schon für Dezember 2021 angekündigt. Nach ersten Hinweisen verfehlte die Substanz eine Signifikanz bei den primären Endpunkten.

*2022, 2023:* Insgesamt können für 2022 und 2023 bei 17 Substanzen Resultate erwartet werden. Bei firmengetriebenen Studien werden die ersten Ergebnisse unmittelbar nach der Analyse zur Information der Investoren bereitgestellt, die medizinische Publikation kann dann Monate später folgen. Ein Beispiel dafür sind die Ergebnisse der Solanezmab- bzw. der EXPEDITION-Studie. Die Pressemitteilung von Lilly zum Studienergebnis erschien sofort nach Abschluss der statistischen Analysen im November 2016 [8], die Veröffentlichung [6] mehr als ein Jahr später im Januar 2018. Hinsichtlich der Studienergebnisse können also Pressemitteilungen weitaus aktueller sein als die Datenbank.

Eine Limitation der Betrachtung ist, dass Studien zur Alzheimer-Erkrankung nicht zwangsläufig auf www.clincaltrials.gov eingetragen werden müssen, sodass es sein kann, dass lokale und kleinere Studien durch diese Herangehensweise nicht erfasst werden. Allerdings ist für die Zulassung eines Medikamentes eine aussagekräftige Phase-III-Studie nötig.

Das Ergebnis ist von den verwendeten Suchbegriffen und dem Zeitpunkt der Suche abhängig. Abgeschlossene („completed“) oder beendete („terminated“) Studien wurden hier nicht berücksichtigt. So tauchen beispielsweise die Studien zu Aducanumab (EMERGE und ENGAGE) der Firmen Biogen und Eisai nicht mehr in der Tabelle auf, können aber nach Eingabe entsprechender Suchkriterien in der Datenbank gefunden werden.

Weiterhin kann nicht garantiert werden, dass die in der Datenbank vorhandenen Daten alle aktuell sind. Gegebenenfalls können Angaben im Internet oder der Literatur weitere Informationen liefern.

Trotz der genannten Einschränkungen erlaubt die Datenbank www.ClinicalTrials.gov eine differenzierte Suche und gibt einen raschen Überblick über die derzeit stattfindenden Studien.

## Fazit für die Praxis

- In den kommenden Jahren kann mit einer Reihe von Ergebnissen zur medikamentösen Behandlung der Alzheimer-Erkrankung gerechnet werden.
- Die meisten Studien zielen auf eine den Krankheitsverlauf verändernde Behandlung ab.
- Auf die auf Amyloid und Tau wirkenden Substanzen, insbesondere auf die monoklonalen Antikörper, konzentrieren sich die größten Erwartungen.
- Kurz vor der Zulassung stehende Substanzen lassen sich derzeit nicht identifizieren.
